# Beyond the Image Frame: An Art-Based Pedagogical Framework for Teaching Diagnostic Reasoning in Breast Ultrasound to Medical Students

**DOI:** 10.3390/diagnostics16040642

**Published:** 2026-02-23

**Authors:** Marcin Śniadecki, Maria Morawska, Patrycja Kijańska, Olga Kondratowicz, Julia Nowakowska, Oliwia Musielak, Abhishek Singla, Ritu Amit Chhabria, Hanaf Alvi, Amelia Banaszak, Lena Grono, Diana Akhmed, Klaudia Kokot, Maksymilian Grzelak, Konrad Duszyński, Katsiaryna Marozik, Patrycja Jaworska, Jakub Majchrzak, Natallia Krupovich, Zuzanna Boyke, Julia Respondek, Weronika Ciećko, Ewa Bandurska, Jakub Szałek, Agata Rutkowska, Martyna Danielkiewicz, Patryk Poniewierza, Ewelina Klimik, Jarosław Meyer-Szary, Cynthia Aristei, Anna Malitowska, on behalf of Senological Gynecology Working Group

**Affiliations:** 1 Division of Gynecology and Obstetrics, Department of Gynecology, Obstetrics and Neonatology, Medical University of Gdańsk, 80-214 Gdańsk, Poland; marysiamorawska@gumed.edu.pl (M.M.); patrycjakijanska@gumed.edu.pl (P.K.); olgakondratowicz@gumed.edu.pl (O.K.); julia.nowakowska@gumed.edu.pl (J.N.); a.abhisingla@gumed.edu.pl (A.S.); rituchhabria@gumed.edu.pl (R.A.C.); hanaf.alvi@gumed.edu.pl (H.A.); ameliabanaszak@gumed.edu.pl (A.B.); lenagrono@gumed.edu.pl (L.G.); diana.akhmed@gumed.edu.pl (D.A.); klaudia.kokot@gumed.edu.pl (K.K.); maksymilian.grzelak@gumed.edu.pl (M.G.); kduszynski@gumed.edu.pl (K.D.); marozik.katsiaryna@gumed.edu.pl (K.M.); patrycja.jaworska@gumed.edu.pl (P.J.); majchrzakjakub@gumed.edu.pl (J.M.); nataszakrupowicz@gumed.edu.pl (N.K.); agata_rutkowska@gumed.edu.pl (A.R.); mmdanielkiewicz@gumed.edu.pl (M.D.); 2 Outpatient Gynecologic and Senologic Clinic “Wolf”, Memorial MD Zofia Garlicka Clinica Femina Centra—Polyclinic of Gynecology and Senology, 80-032 Gdańsk, Poland; 3 Department of Surgical Oncology, Transplant and General Surgery, Faculty of Medicine, Medical University of Gdańsk, 80-214 Gdańsk, Poland; omusielak@gumed.edu.pl; 4 Department of Art History, Faculty of History, University of Gdańsk, 80-308 Gdańsk, Poland; z.boyke.073@studms.ug.edu.pl; 5 Graphic Design Department, Faculty of Graphic Arts, Academy of Fine Arts in Gdańsk, 80-836 Gdańsk, Poland; julia.respondek@akademia.gda.pl; 6 Center for Competence Development, Integrated Care and e-Health, Medical University of Gdańsk, 80-214 Gdańsk, Poland; weronika.ciecko@gumed.edu.pl (W.C.); ewa.bandurska@gumed.edu.pl (E.B.); 7 Faculty of Social Sciences, Pontifical University of John Paul II in Kraków, 31-002 Kraków, Poland; szalek.management@gmail.com; 8 Faculty of Medicine, Łazarski University, 02-662 Warsaw, Poland; patryk.poniewierza@lazarski.edu.pl; 9 Department of Product Design, Faculty of Design, Strzemiński Academy of Fine Arts in Łódź, 91-726 Łódź, Poland; ewelina.klimik.asp@gmail.com; 10 Department of Pediatric Cardiology and Congenital Heart Defects, Medical University of Gdańsk, 80-214 Gdańsk, Poland; jaroslaw.meyer-szary@gumed.edu.pl; 11 Radiation Oncology Section, Department of Medicine and Surgery, University of Perugia and Perugia General Hospital, Sant’ Andrea delle Fratte, 06-156 Perugia, Italy; cynthia.aristei@unipg.it; 12 Department of Ethics, Adam Mickiewicz University, 60-568 Poznań, Poland; anna.malitowska@amu.edu.pl

**Keywords:** breast cancer, senology, slow education, diagnosis, Renaissance paintings, interpretation, symptoms, time poverty

## Abstract

Breast ultrasound is a key diagnostic method for breast cancer and relies heavily on the interpretation of visual cues. At the same time, medical education is increasingly being driven by time constraints, which favors rapid pattern recognition, limiting the scope for reflective image analysis and the diagnostic process. Therefore, the aim of this study was to propose and evaluate an artistic and pedagogical teaching model, inspired by the interpretive practices of Italian High Renaissance painting, as a tool to support the development of diagnostic reasoning in breast ultrasound. This model focuses on careful observation, analysis of the relationship between detail and the overall image, and the conscious transformation of visual cues into clinical meaning. This study was conducted during the four-day ARSA Think Tank Meeting (ARSATTM). Medical students worked in four groups; two groups received methodological training based on visual cue analysis, and two did not. All groups performed identical tasks involving the interpretation of breast ultrasound images and ultrasound examinations on real patients. The results indicate that an artistic–pedagogical teaching model to promote more coherent and reflective diagnostic reasoning in breast ultrasound is feasible. Therefore, integrating this approach may be a valuable addition to medical students’ ultrasound education in the realities of limited clinical time.

## 1. Introduction

**Figure 1:**
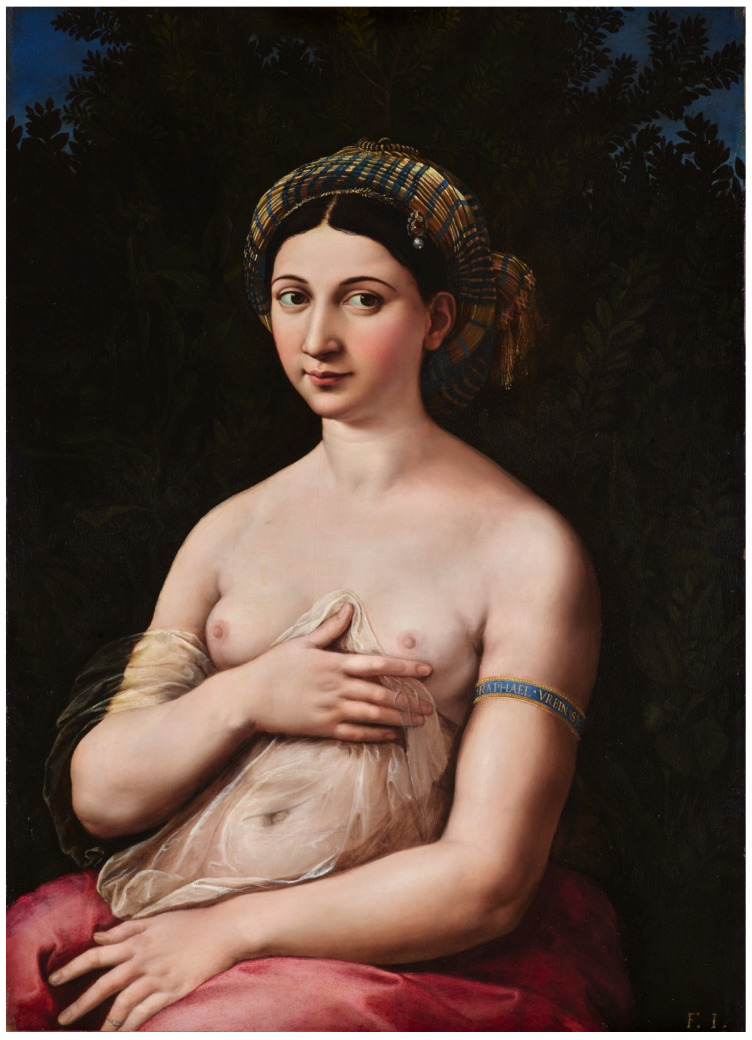
A Portrait of a Young Woman (called “La Fornarina”), (1518–1519), oil on wood panel, *Raffaello Sanzio da Urbino* (1483–1520). In: Galleria Nazionale d’Arte Antica in Rome (reproduced with permission).

In the years 1518–1519, Raffaello Santi *detto* Sanzio (1483–1520), also known as Raphael, painted *The portrait of a Young Woman (La Fornarina)*, a portrait of his love, Margherita Luti (1500–1522), exemplifying a timeless beauty and archetypal love between artist and his muse (Figure 1) [1]. In the painting, Margherita is depicted holding a veil up to her exposed breasts and, with the index finger of her right hand, gestures toward her left breast and axilla, possibly suggesting the presence of a lateral oval mass [2]. Although the suggested “diagnosis” of a breast deformity—interpreted as left-sided breast cancer—is challenging, this ambiguity may offer a valuable foundation for educational research and methodology of scientific studies [3,4].

According to recent data from The Global Cancer Observatory, breast cancer (BC) is the most frequent malignancy worldwide, accounting for 2,308,897 new diagnoses in 2022, for which the most important risk factor is a female sex [5]. Furthermore, the incidence of breast cancer is increasing at a rate of 1% per year in high-income countries. This phenomenon is believed to be attributable to various risk factors, including increased body mass, nulliparity, and the age of the mother at the time of the first birth of a child, with the majority of births occurring after the age of 30 [6]. Concurrently, the mortality rate for BC remains high (6.9%), ranking fourth globally among all 36 cancer sites [6,7].

The prevailing notion suggests that contemporary society is experiencing a state of “time poverty”, characterized by the scarcity of sufficient time to thoroughly evaluate choices. This concept is further elaborated on in the following assertion: the practice of “time hoarding” contributes to a deficit in personal time management [8]. In the healthcare sector, “time poverty” is of critical importance, as effective decision-making at various levels is essential for the health and well-being of individuals and the functioning of society as a whole. The concept of “slow medicine” is inextricably linked to the broader philosophy of “slow life”, a term that encompasses a variety of approaches to life and its various branches [9]. In summary, the definition of “slow medicine” proposed by Pieter Cohen and Michael Hochman, the pioneers behind the series of discussions at the University of Cambridge entitled “Updates in Slow Medicine”, characterizes the practice as prioritizing the patient treatment over disease treatment [10]. The pedagogy employed in the training of medical students is congruent with the principles of “slow medicine”. It is imperative to consider the implications of this matter, as it pertains to the field of oncology. The COVID-19 (Coronavirus disease 2019) pandemic disrupted one of the foundational tenets of oncology: the personalized care of every cancer patient. Yet, the necessity of triaging care, such as determining when observation alone is sufficient in the face of reduced testing and limited in-person visits, emerged as a potential benefit. This adaptation can be viewed as a pragmatic application of slow medicine [11,12,13,14].

In the context of breast cancer, visual signs play a pivotal role in the diagnostic process, given that the diagnosis of this condition is predominantly based on imaging modalities. Here, “imaging” is understood in a broad sense, ranging from traditional imaging modalities such as ultrasound to pathological assessment through color-coded protein expression (e.g., immunohistochemistry), and increasingly to non-invasive molecular diagnostics [15]. However, the roots of medical imaging can be traced back to the Italian High Renaissance, a period in art history characterized by the fusion of Art and Science [4]. During this era, naturalism—the depiction of reality in visual artistic works—emerged as the predominant characteristic of the Italian High Renaissance [16]. The contemporary trend of inventing “diagnostic images”, termed *iconodiagnosis*, has emerged from the faith of those artists [17,18].

This perspective of contemporary daily work often shows the methodical approach, which, in contrast to the methodological one, utilizes scientific methods while developing standards in a non-reflexive manner [18,19,20,21]. In this article, we document our reflective engagement with both methodical and methodological scientific cultures. We pose two integral questions: Can more be done in the realm of breast cancer prevention? And can this reflective process be meaningfully integrated into the education of medical students? In light of the increasing integration of Artificial Intelligence (AI) into our professional landscape, it is imperative to utilize the “slow movement” and methodological criticism in medicine. The central goal of this pedagogical protocol was to propose how medical students can be taught today in line with the slow approach, and this will directly influence the quality of their future teaching of AI. We believe that this way of teaching will be more meticulous and, consequently, will require a longer time frame but will be more effective.

## 2. Methods

This work is a qualitative educational study that focuses on students’ interpretation of diagnostic signs. The aim of this work is not to evaluate the effectiveness of teaching in quantitative measures, and the comparison of groups is descriptive. The authors intended that the student workshops (non-randomized in the basic protocol) on medical research would highlight the interpretive functions of diagnostic tests, comparing medical diagnosis (and interpretation in the natural sciences) with the interpretation of works of art.

In particular, we aimed at the way of formulating diagnostic answers (from methodical, iterative to methodological, interpretative), the change of the language of description (from a simple diagnosis to an attempt at interpretation), and considering the patient’s context.

**Figure 2:**
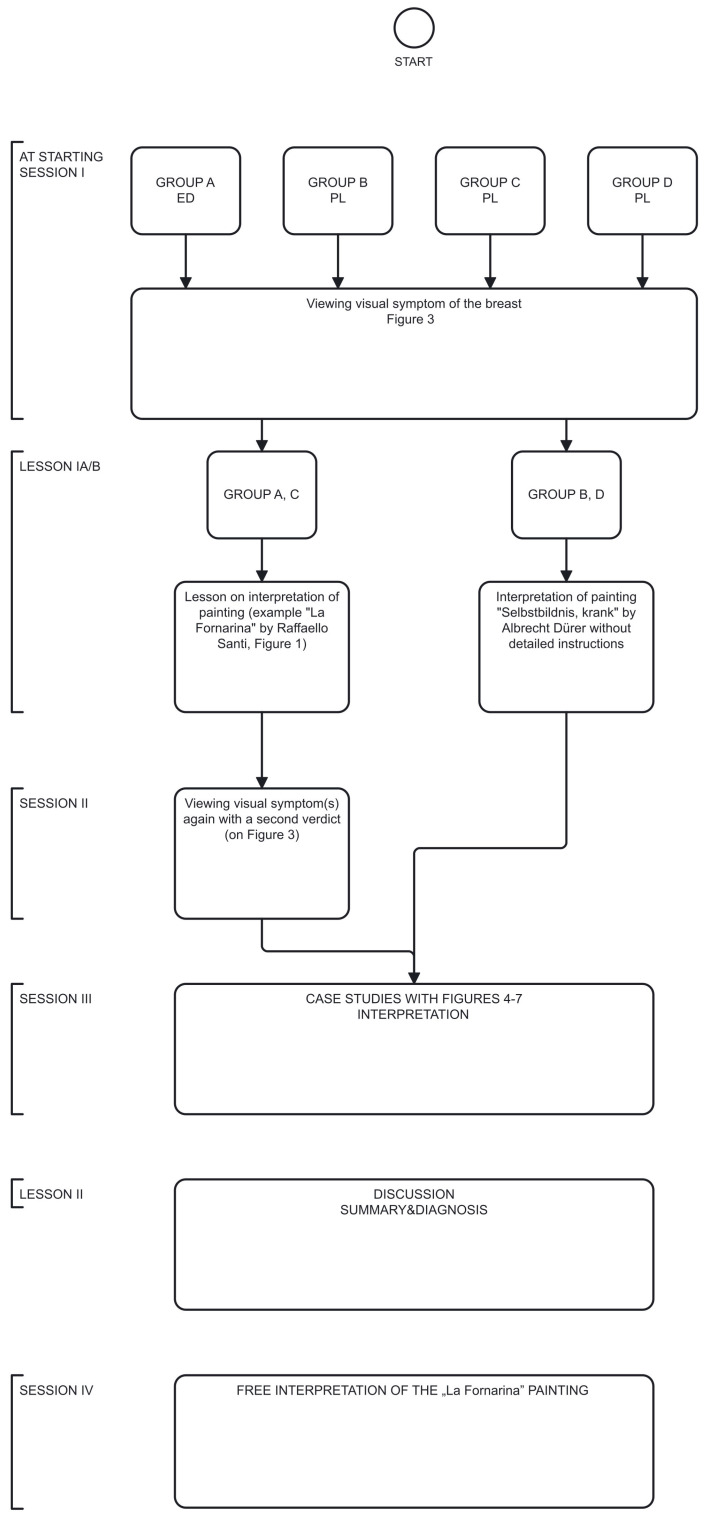
Design of the meeting.

Four groups of medical students participated in four sessions according to the basic, non-randomized protocol; three of the groups were Polish-speaking, and one was English-speaking (Group A—English Division (ED): A.S., P.K., H.A., R.A.C., Group B—Polish Division (PD): L.G., A.B., O.K., J.N., Group C (PD)—K.D., K.M., P.J., J.M., N.K., Group D (PD): M.M., D.A., K.K., M.G.), all co-authors of this study (Figure 2).

**Figure 3:**
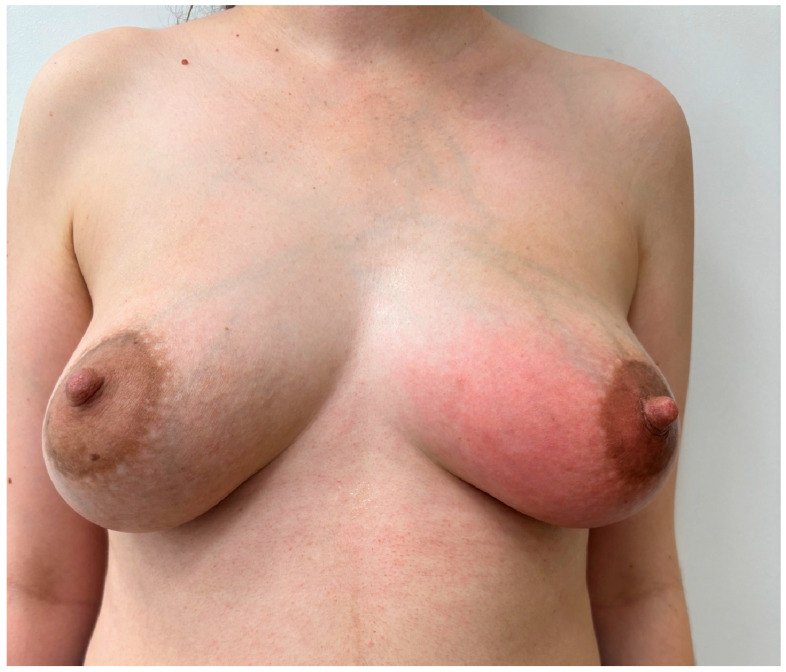
A female patient with redness of the skin of the left breast.

**Figure 4:**
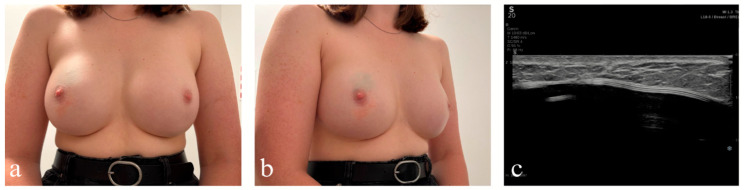
A 24-year-old patient presenting to Group A; (**a**) front view of the patient’s breasts; (**b**) side view of the breasts, with the bruising and redness of the right breast more clearly visible (see description in the text); (**c**) ultrasound image of the bruised area of the right breast, with the implant present (BI-RADS 2).

**Figure 5:**
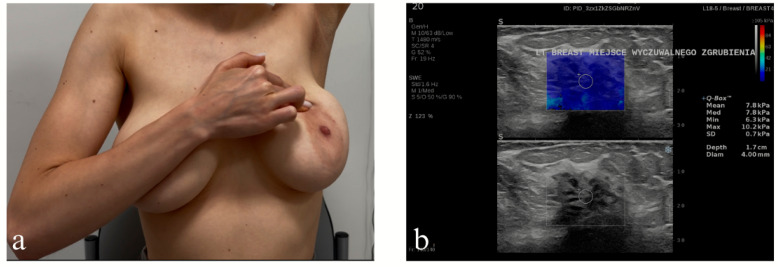
An 18-year-old female patient presenting to Group B and examining her breasts in breast self-examination; (**a**) the patient points to a palpable lesion in her left breast (still from the self-examination); (**b**) the lesion turns out to be a non-focal, non-mass like lesion (NML).

**Figure 6:**
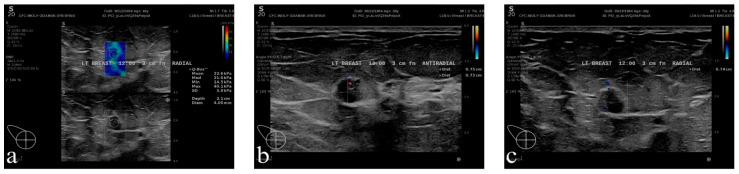
A 40-year-old female patient’s ultrasound pictures presenting to Group C; (**a**) elastography presentation of palpable area in the left breast (soft lesion), (**b**) B-mode and microflow imaging presentation of the identified lesion, anti-radial probe setting, (**c**) B-mode and microflow imaging presentation of the identified lesion, radial probe setting.

**Figure 7:**
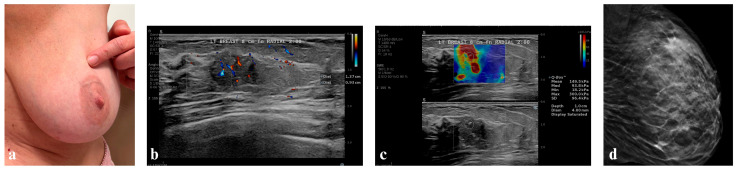
A 43-year-old female patient of Group C; (**a**) location of palpable tumor in the left breast, (**b**) B-mode presentation with microflow imaging showing the size of the lesion, (**c**) elastography presentation of palpable area in the left breast (hard lesion), (**d**) lateral aspect of the left breast at MMG-tomosynthesis.

I **At starting session (I session).** In the initial session of the four-day workshop, all four groups (A–D) of students (Group A on day 1, Group B on day 2, etc.) were independently provided with the same clinical image of the patient with left breast abnormality that had been retrieved by the project developers (Figure 3) and assigned the task to formulate a medical diagnosis in the form of a single sentence within 5 min.
II **Lesson Ia** on the interpretation of the painting *La Fornarina* by Raffaello Sanzio. This painting was used as an example of a painting with “diagnostic” properties in cases of **Groups A and C** but not in cases of Groups B and D. The selection of this painting for analysis was motivated by the controversy surrounding its diagnostic utility and its continued citation as a foundational reference for various medical considerations [3,4,17,22]. During this lesson, a methodology for interpreting the “medical” image from the perspectives of visual culture, art history, and visual arts was presented (Supplementary Material S1).
III **The second (II) session.** During the second session, **Groups A and C**, who had received the Lesson Ia on image interpretation, were given tasks of issuing a diagnostic verdict again.
IV **Lesson Ib.** Students in **Group B and D** interpreted another Renaissance artwork in the form of a drawing by Albrecht Durer entitled *Selbstbildnis, krank* but without detailed instructions driven by art history and semiology [23]. Students from groups A and C did not participate in this activity.
V **In the third (III) session,** each group of medical students (Groups A, B, C, and D) displayed a case study with clinical images of four different patients: Group A—Figure 4, Group B—Figure 5, Group C—Figure 6, Group D—Figure 7. The students were then asked to formulate an interpretation of all signs and symptoms they see.
VI **Lesson II** was a summary of the previous sessions and lessons, including an explanation of the diagnosis.
  After this lesson, each group of students underwent a “Be like Raphael” workshop (Supplementary Material S2).
VII **During the fourth (IV) session**, students were tasked with creating their own interpretation of Raphael’s *La Fornarina* painting based on the specific case studies they analyzed in the previous session. They were given one week to complete this task. This session was conducted fully online. A summary of the proceedings and results of all sessions is provided in the Results section of this report and in the Supplementary Material S3 as well.

The medical interpretation lesson for medical students was developed in March and April of 2025. Its principles were first presented at the ATINER (Athens Institute) conference in Greece on 6 May 2025 [22]. The “Beyond the frame” meeting was conducted during June 2025.

## 3. Results

### 3.1. At Starting Session—Initial Student Interpretation

In the first session, students were given the task of diagnosing the condition based solely on the photograph (Figure 3) without any introduction. Group A decided to diagnose “inflammatory breast cancer”. Group B concluded: “Unilateral redness, vasodilation, swelling, and tension in the right breast suggest inflammatory breast cancer.” Group C diagnosed briefly: “Inflammatory cancer or mastitis.” Group D concluded that it was “inflammatory BC due to the extensive redness and retraction of the nipple.” All four groups of students provided similar responses, albeit of varying lengths, including justification for the diagnosis (groups B and D).

### 3.2. Lesson Ia—Seminar Room (Groups A and C)

According to C.H. Espinel, the breast deformity portrayed by Raphael in the painting *La Fornarina* (Figure 1) may represent the signs of breast cancer: mass, retraction, skin discoloration, enlarged lymph nodes, and arm swelling. The symptoms seem to be so precise that we are inclined to make a simulated diagnosis and even try to determine the stage of the disease [2]. In Supplementary Material S1, we showed the introduction to interpretation of paintings [24,25,26,27,28,29,30].

Using semiology as interpretation methodology aimed at transferring signs into meanings in medical context, there are three types of signs—indexical one (it represents real, physical connection between the signifier and the signified), iconic one (it represents an object if the sign and the object are similar), and symbolic one (there is a convention that assigns denotations to them, which means that symbols require interpretation based on specific conventions) [30,31].

Table 1 provides an explanation of the signs in *La Fornarina* painting from the perspectives of medical and artistic–visual culture, according to the authors mentioned above, Bettini, Rose, and Peirce, among others [25,30,31]. Of the three types of signs that can be identified in the painting, most of the meanings relate to the symbolic layer of the image. *La Fornarina* is a wedding portrait. The bare breasts, laurel, myrtle, quince, and a pearl on the turban are all associated with the theme of marriage. The figure is both sensual and innocent. The bracelet with Raphael’s name on it is a proof of love. In the case of Raphael and his muse, according to art historians and Raphael’s contemporary, Giorgio Vasari himself, it was also about the forbidden and impossible love between Raphael and Margherita Luti [1].

### 3.3. Second Session

Groups B and D were not asked to add any new observations or conclusions. Group A posed more of a question than a diagnosis: “Ductal mastitis or maybe some oncological inflammation?”

Group D said: “We would differentiate this from breast inflammation, especially if the woman was lactating.”

The introduction in art interpretation therefore sowed doubts in Groups A and C. Groups B and D had to stick to their positions (they had no other option).

### 3.4. Lesson Ib—Seminar Room (Groups B and D)

Raphael’s painting is not the only work from this period that attempts to present in detail at least supposed medical symptoms for analysis. Around 1509–1511, Albrecht Dürer drew a self-portrait, which he used as a note to send to his doctor, describing his symptoms [32]. In this painting the artist looks directly at the viewer, but our attention is directed to the artist’s finger pointing to his left side where an outlined patch of yellow has been drawn, and the artist’s German inscription: *Do wo der gelb fleck ist und mit dem finger drawff dewt do is mir we* (“There, where the yellow spot is located, and where I point my finger, there it hurts”) [23,32,33]. The choice of color was not accidental and was probably added for unequivocal emphasis. During the Renaissance, a well-known practice was to share works of art remotely, not just those containing symptoms (for example the preparatory sketches). There is evidence that Dürer gave his engravings to Raphael. Therefore, the choice of Dürer as the artist-exemplar here is deliberate [34].

### 3.5. Third Session

After showing students photos of patients, the instructor asked about the history of each patient without drawing any conclusions beforehand. The purpose of this session is to present cases to the students. Each description includes a diagnosis. The examination was performed using a HOLOGIC Supersonic Mach 20 device with an L18-5 probe, using B-mode, Doppler, AngioPlus, and shear wave elastography techniques (Marlborough, MA, USA).

#### 3.5.1. Patient of Group A

Case presentation: K.B., a 24-year-old female patient, was presented with bruising at 12 o’clock on the upper outer quadrant of the skin of the right breast (Figure 4a,b) to the university outpatient clinic. During the interview, she reported a lesion to a gynecologist for a senological consultation after her partner palpated a lump in the upper outer quadrant of her right breast. It was her second breast examination, because due to the high genetic risk (there were already cases of breast cancer in the patient’s family—mother, aged 51, and aunt, aged 30) she started to have regular BUS examinations in a private setting.

The gynecologist, after performing a breast palpation, suspected fibroadenoma and referred the patient to a radiologist for BUS. The radiologist gave the lump BI-RADS-3 category but ordered a core needle biopsy due to the family history of breast cancer. The biopsy showed the presence of luminal B breast cancer subtype. The VUS type mutation was detected in the BRCA 1 gene. A bilateral (at the patient’s request) mastectomy with reconstruction was performed, complicated by postoperative infection requiring local treatment, and subsequent treatment with goserelin and tamoxifen was introduced. Due to the COVID-19 pandemic, the patient communicated several times with the oncologist by email (e.g., regarding the transfer of paraffin blocks for the Oncotype Dx Breast Recurrence Score^®^) and by phone with the surgeon after noticing disturbing symptoms of inflammation after mastectomy, getting immediate help every time. She studies computer science but is very physically active.

Physical examination: Breasts were symmetrical, with implants, with no nipple discharge nor skin changes but a blue lesion on the upper outer quadrant of the right breast. Additionally, at the lower outer quadrant of the right breast was a red round skin lesion.

Ultrasound examination: Breasts with a fatty structure, without focal changes, with normal lymph nodes (Figure 4c).

*Interpretation of Group A:* The blue discoloration on the right breast is caused by methylene blue, a blue dye. The red discoloration is caused by chafing due to dry skin rubbing against the climbing wall during climbing.

Effect: This case taught students how to understand the medical history that may underlie visual symptoms of skin discoloration.

#### 3.5.2. Patient of Group B

Case presentation: M.K., an 18-year-old female patient, was qualified and enrolled in another study involving remote monitoring of patient compliance with monthly breast self-examination (BSE) in a university outpatient setting. This monitoring was performed regardless of the patient’s medical knowledge or whether the patient had received medical training, following an initial breast ultrasound examination (results not yet published). Utilizing a smartphone, the patient informed the study leader (M.Ś) that she had perceived a barely perceptible hardening in her left mammary gland (see Figure 5a). This alteration was not evident in the previous month.

Physical examination: A barely perceptible change in the area indicated by the patient.

Ultrasound examination: The ultrasound examination revealed a structural abnormality, a non-mass lesion (theoretically impossible to assess on the BI-RADS scale) (Figure 5b).

*Interpretation of Group B:* To underline the physiological nature of the palpable lesion, a grade of 1 was assigned in the BI-RADS category.

Effect: The young patient’s breast self-examination sign is visually reflected in shades of gray during an ultrasound examination (here: tissue stiffness assessment). The case demonstrates that one symptom in one study is reflected in another study.

#### 3.5.3. Patient of Group C

Case presentation: The patient G.Ż.-S. is a 40-year-old female who presented with tenderness in the left breast in the armpit area to the BUST. The woman in question has two children, both of whom she breastfed for a total period of 22 months. She currently wears a bra size of 85G and considers her breasts to be large. The patient’s medical history does not include any oncological or general diseases. The patient does not perform BSE.

Physical examination: No changes were visible on examination, and no palpable changes were found during the examination.

Ultrasound examination: In the left breast, a solitary focal lesion measuring 7.4 × 7.4 × 7.5 mm was identified in the upper outer quadrant, situated approximately 3 cm from the nipple. The lesion exhibited an irregular outline, a soft consistency on elastography, and vascularization features, corresponding to BI-RADS category 4a (Figure 6a–c). Furthermore, the presence of blurred structural lesions and dilation of the milk ducts to 3 mm was observed. In the right breast, simple cysts and NMLs corresponding to BI-RADS 2 were observed, with ducts dilated to 5 mm. A thorough examination of the lymph nodes on both sides revealed no evidence of pathology.

*Interpretation of Group C:* The palpable lesion in the left breast required verification with age-appropriate imaging; this verification, in turn, required the use of all basic imaging features—multiparametric and multiplanar—to identify the lesion and determine whether it was suspicious or not.

Histopathology: Examination revealed luminal A breast cancer in three foci measuring 2–3 mm each with the tumor.

Effect: A lesion in the mammary gland must be viewed in at least two planes on an ultrasound examination, along with additional visual aids such as Doppler or elastography. Conclusions cannot be drawn based on a single scan. Even with a modern visual examination such as ultrasound, we cannot draw far-reaching diagnostic conclusions based on the entire examination (unlike iconodiagnosis)—a biopsy is necessary.

#### 3.5.4. Patient of Group D

Case presentation: Patient A.F., a 43-year-old female, with a palpable lump in the upper outer quadrant of her left breast (around 3:00 o’clock) was presented to the senological unit (BUST). During a previous ultrasound breast examination, it was assessed as BI-RADS-1. The patient had her first menstruation at the age of 14. The patient had two children and breastfed for about 48 months. BMI was within the normal range (20.96 kg/m^2^), bra size 80D—the patient subjectively assesses her breasts as large (Figure 7a). She tries to regularly examine her breasts with BSE and BUS.

Physical examination: Breasts were symmetrical, no nipple discharge, no skin changes.

Ultrasound examination: Left mammary gland with fatty-glandular structure, palpable by the patient at 2 o’clock, 8 cm from the nipple, there is a poorly defined area, well vascularized, calcified, with a stiffness of >85 kPa (Figure 7b,c). Initially, during the first BUS up down scanning (see description of the workshop, Supplementary Material S2), the lesion was overlooked, “dissected” better after second-look side-side and after patient instructions (the lesion in the supine position was arranged peripherally). Milk ducts and lymph nodes appeared normal. The right mammary gland was of fatty-glandular structure with no alarming changes. Milk ducts and axillary lymph nodes were normal. In conclusion, the tumor was categorized as a BI-RADS-4c lesion, initially considered a non-mass lesion, currently suspected high-grade DCIS or luminal-type cancer in the left breast at the pre-diagnostic level, for urgent further evaluation by mammography with tomosynthesis (Figure 7d).

*Interpretation of Group D:* A large breast is difficult to examine, but it is possible to detect a change if the patient undergoes frequent examinations and knows her breasts. Ultrasound, following the indication, can reveal what the patient is indicating and help guide the diagnosis.

Histopathology: Examination revealed luminal A breast cancer with a concomitant component of solid-cribriform DCIS of intermediate grade of cellular atypia and with microcalficifications. Invasive cancer is within around 80% of the mammotomy specimen.

Effect: As in the previous cases, we combine palpation with ultrasound imaging and tomosynthesis to emphasize the impossibility of drawing diagnostic conclusions without a history and thorough examination, the results of which are subject to our discerning observation and interpretation.

### 3.6. Conclusions

#### 3.6.1. Summary

All groups made similar interpretations given the opportunity to explore the entire case, starting with a live patient. Despite the fact that groups B and D were “cut off” from the interpretation lesson and were only given a semblance of insight into the art (a painting by Dürer without art–historical commentary), they performed similarly. The only differences were visible in groups A and C before and after the interpretation lesson. This may indicate that, despite suggestions of a possible diagnosis, the student groups are attentive and resistant to artistic intervention, trying to avoid diagnostic errors when meeting the patient.

#### 3.6.2. Discussion

Every great artist had his students. Like Perugino and his student Raphael, and the latter’s younger colleague and heir Giulio Romano, medical students—“the modern-day Romanos”—are the heirs of the masters and learn that to get closer to the diagnosis (the nature of the disease), they must first understand the principles of creating an ultrasound image. The creation process is similar to how masters and students jointly created major artworks, which the students then copied, developing their own style [34]. Considering educational reasons, this approach makes students aware that the diagnosis cannot be made “at first glance” but depends on their methodological approach and cognitive skills, allows them to participate directly in the process of creating the test result, and develops a sense of responsibility for its quality. Supplementary Material S2 presents a brief instruction on how to operate an ultrasound probe and documents the lesson in which students participated during the meeting. Teaching imaging based on the paintings of the Italian High Renaissance is a new educational approach, and it is in opposition to iconodiagnosis, which assumes the possibility of diagnosis based on a single image, even if the assessment of a painting seems to be surrounded by semiotics and art history [18].

To show the rationale for our approach and understand the parallel between the painter and the sonographer, a minimum knowledge of Renaissance painting using new discoveries such as the possibility of shaping figures with oil paints or the use of mathematical principles of perspective allowing for the expansion of the image in the literal and figurative sense of the word is enough. Raphael applied at least nine layers of pigment and glazes of the left breast of *La Fornarina* [2]. In fact, modern ultrasonography distinguishes up to 256 shades of grey, and each pixel of the image has a specific brightness on that greyscale from 0 (black) to 255 (white). The multi-parameter assessment of focal lesions in US breast examination consists of anatomical mapping of the structures of the breast in grey scale and the superimposed effects—Doppler, microflow imaging in the tumor, and elastography. The latter, which maps the elastic properties and stiffness of soft tissue that result in breast deformations, is the closest to the methodology of painting in its application of slight pressure with the US head on the area of the breast being examined. For the five standards of strain rate that have been adopted, and calculation of the strain ratio or directly measured tissue stiffness in kilopascal units, elastography is useful in refining the ultrasound classification of breast lesions according to the BI-RADS, especially in grades 3 and 4 [35]. These elements of the contemporary sonologist’s toolkit complement each other, as do the oil colors and glazes of the Renaissance painter’s palette. To obtain the final tumor depiction in the ultrasonography, the US operator’s procedure involved 11 steps, similarly to the painter’s one. Table 2 presents parallels between artistic images and ultrasound images.

Students must know that common imaging modalities, such as ultrasound (US) or mammography (MMG), address the knowledge gap that remains after visual and physical examinations and increase the sensitivity of breast cancer detection in screening tests by at least 40% compared to the detection rates of clinical breast examinations alone [36,37]. Concurrently, this diagnostic refinement, which attain nearly 100% detection rates of suspected lesion in the breast when two imaging techniques are combined, is due to the BI-RADS descriptive lexicon for lesions found in the tumor image [38]. On the other hand, it is imperative to acknowledge the limitations of the BI-RADS modalities (US, MMG, magnetic resonance imaging, MRI) in accurately detecting breast masses that are subsequently confirmed to be malignant by final histopathological evaluation (Table 3) [39,40,41,42,43,44].

### 3.7. Fourth Session

In this final session, which concluded the entire meeting, students were given a week to complete the task of freehand interpretation of “Contemporary *La Fornarina*,” the protocol from this part is provided in the Supplementary Material S3. Student work demonstrates the learning process and is not a measure of teaching effectiveness.

## 4. Final Discussion

Proper interpretation of the test results and selection of the appropriate diagnostic or therapeutic procedure require careful clinical and radiological assessment, considering a thorough medical history, detailed physical examination, and the results of all other diagnostic tests.

In this breast ultrasound workshop, which involved several groups of medical students, the interpretative function of diagnostic testing was the focal point. The emphasis was placed on the pivotal role of interpretation in medicine, underscoring the necessity of evidence-based medicine.

After the lesson on the interpretation of signs, students were presented with additional ultrasound images of the breast. They are not instructed to interpret the images but rather inquire about them. They then used their intelligence to interpret cases and further develop their interpretative skills. A breast ultrasound workshop was also held for students, who had the opportunity to take part in performing a live breast ultrasound examination.

The effects of the intervention, which assumed that two groups received more background for interpretation while the other two groups did not, were not apparently visible. This may be explained by the small number of students, but each group included several individuals with varying knowledge, critical approaches to research, sensitivity to art, and empathy. Therefore, the verdicts of the groups could be similar. We cannot rule out the possibility that some criticism of the rapid diagnosis was voiced at the beginning, in the At Starting Session. The students were not recorded. However, it can be noted that initially, the groups were under some pressure from the meeting, which supposedly suggested a cancer diagnosis based on a single image.

Regardless of the type or stage of the ailment portrayed, attempts to represent visible symptoms and thereby diagnose an ailment (even remotely) are indicative of a contemporary era and a continuation of a Renaissance phenomenon [45,46]. Currently, though images can be used to significantly substantiate support for a diagnosis, the diagnosis itself is not yet technologically possible using images alone [47]. Our documented workshop-discussion panel is the first example of its kind to develop this claim in an educational context.

Technological advances are also important for improving accessibility. The keys to positive patients’ cancer treatment results are a prompt diagnosis of the patient and acting in the early stages of the disease [48]. The potential for patients to show their own history of symptoms and beyond may be pivotal in achieving enhanced overall outcomes in the treatment of oncological patients [49].

All indications point to the idea that integration of information technology techniques will soon become an integral part of any medical service [50]. We may also assume that in the coming years, BUS will provide improved mapping of tumor structure, and thus tumor biology, by imaging the surface and interior, assessing microflows, minimizing artifacts, and improving imaging resolution, thereby making it comparable to magnetic resonance imaging (especially in 3D volumetric blocks). This opens up previously unknown possibilities of breast examination at home, supported by modern imaging technologies, as an important step towards increasing the availability of breast cancer diagnostics [51,52,53,54].

Contemporary imaging techniques, ranging from US to the attempts of characterization of the molecular biology of cancer from radiographic images involving artificial intelligence (AI) (radiomics), we see notable additional parallels with Renaissance artists’ attempts to provide realistic material in the image they created [45].

In conclusion, this is a lesson of “transfiguration of signs into meanings” concomitant with a medical skepticism, both being useful in education. In our opinion, medical students and young doctors are in a challenging educational situation. They are under pressure from time constraints and growing demands from their environment, including new technologies. AI support will not be utilized to its fullest potential, and it may even be misused, if we do not constantly remind young students of the need for critical and methodological (not methodical) thinking when making diagnostic decisions. Routine and habit cause details of the image to go unnoticed—if we do not know what to look for, but also if we do not know the story that the image tells, which is the story of the patient. This is clearly visible in films such as *The Mill and the Cross* [53] or *Girl With a Pearl Earring*, [54] once we realize that a 20 min sonogram consists of ca. 24,000 individual images (*frames*) (personal communication from the HOLOGIC technician). Fragments of the tumor/patient history can be told with short BUS clips.

## 5. Recommendations

By demonstrating our process of analyzing paintings, we aim to make our students understand how diagnosing is merely observing a given sign objectively. Diagnosing is the interpretation of all information coming from the patient—including that processed in the form of a medical image.

Analyzing disputes between researchers as a clash of methodologies in the way that interpretation transforms signs into meanings is a critical and educational methodological reflection. In this regard, it is worth remembering that the paramount objective of diagnosis is not the disease but the patient—and this is “beyond the frame of an image.”

## 6. Limitations

We are aware that our interpretations are neither absolute nor final. They are dependent on criteria that are never exhaustive, meaning that there are many different methods that can be used to interpret visual representations. Furthermore, we are required to justify interpretation itself as the chosen method for understanding, interpreting and deciphering signs.

This article presents a pedagogical outline of a lesson on the intersection of Renaissance art and ultrasonography, in which non-randomized groups of students are introduced to interpreting painted images in a way that engages them in “imaging” exercises. A weakness of this study is that, despite its length, the lesson is too short to measure the effectiveness of this teaching method. More groups who undergo artwork and medical images interpretation should be involved, and the students’ decision-making abilities should be tested using patient case studies pre and post intervention in the form of an OSCE (Objective Structured Clinical Examination) or another type of evaluation.

## Figures and Tables

**Table 1:** Prototypic explanation of signs from medical [2] and philosophical and visual culture perspectives [1,25,26,27,28,29,30,31] in the painting *La Fornarina* by Raphael as an example.

Type of Signs	Definition	Medical Perspective	Art History/Visual Culture Perspective
Indexical sign	It represents real, physical connection between the signifier and the signified.	The naked breast with a mass, retraction, skin discoloration (deformation)— index sign of breast cancer.	The naked breast—index sign of art nude.
Iconic sign	It represents an object if the sign and the object are similar.	N/A	The art nude is an iconic sign of Western art as visual culture.
Symbolic sign	There is a convention that assigns denotations to them, which means that symbols require interpretation based on specific conventions.	N/A	Raphael uses the female body (*signifiant*) to symbolize sensual beauty and (unfulfilled) love (*signifié*); left side may also symbolize sin: Latin *sinister*—not right.

Legend: N/A—not applicable.

**Table 2:** Comparison of a painter’s and an ultrasongrapher’s steps in the process of creating an image.

	11 Steps to Achieve the Final Portrait of the Left Breast of *La Fornarina*, Including 9 Pigments [2]	11 Steps to Achieve the Final 2D Ultrasound Tumor Image
1.	Applying primer under the paint	2D enhancement: sets the brightness of the entire 2D image
2.	Sketching the outline of the shape	Penetration depth: reduces or increases the size of the 2D image
3.	Black	TGC correction sliders changes the gain at certain penetration depths in the 2D projection, thus allowing for accurate compensation of echo weakening over the time (depth)
4.	Gray	Frequency range: allows the operator to quickly adjust settings for high resolution/low penetration, medium resolution/medium penetration, low resolution/high penetration for a 2D image
5.	Violet	Harmonic imaging: provides better grayscale contrast compared with the standard ultrasound imaging
6.	Blue	Simultaneous multi-frequency operation: this function combines the use of low frequency to increase penetration and higher frequency to maintain high resolution
7.	Pink	Code excitation improves image resolution and penetration of deeper areas
8.	Creamy	Cross-beam imaging (XBeam-CRI): increases contrast resolution with better tissue differentiation and clearer organ contours
9.	Brown	Noise reduction imaging (SRI II): smooths the final image
10.	Taupe	Color Doppler application
11.	Umbra	Use of elastography

**Table 3:** Average tumor size of the mammary gland detected by the most frequently used diagnostic modalities.

Diagnostic Method of a Breast Tumor	Average Tumor Size (cm)	Reference
Magnetic resonance imaging	0.7	[39]
Ultrasonography	1.0	[40]
Mammographic screening	1.4	[41,42]
Clinical breast exam and self-examination	2.05 and 2.1	[43]
Incidental detection	2.85	[44]

## Data Availability

All data regarding this meeting are held by the authors and can be made available upon request.

## Supplementary Materials

The following supporting information can be downloaded at: https://www.mdpi.com/article/10.3390/diagnostics16040642/s1, Supplementary Material S1. The narrative methodological approach of interpreting Italian High Renaissance Paintings. Supplementary Material S2. “Be like the Raphael”—breast ultrasound examination presentation. Supplementary Material S3. Free interpretation of the image of *La Fornarina* by Students.

## Abbreviations

AI	Artificial intelligence
ARSA	Arthur Schopenhauer Research Academy
ARSATTM	ARSA Think Tank Meeting
BI-RADS	Breast Imaging-Reporting and Data System
BSE	breast self-examination
BUS	Breast ultrasound
BUST	Polyclinic of Gynecology and Senology, Division of Breast Ultrasound and Senological Treatment
COVID-19	Coronavirus disease 2019
GLOBOCAN	The Global Cancer Observatory
MMG	Mammography
MRI	Magnetic resonance imaging
NML	Non-mass lesion
SGWG	Senological Gynecology Working Group
US	Ultrasound
