# Supplementary material for: Beyond the Image Frame: An Art-Based Pedagogical Framework for Teaching Diagnostic Reasoning in Breast Ultrasound to Medical Students
Source: Diagnostics (Basel). 2026 Feb 23;16(4):642. doi: 10.3390/diagnostics16040642 (PMC12939138; doi:10.3390/diagnostics16040642)
Supplement: Supplementary file 1 [file diagnostics-16-00642-s001.zip › Supplementary Material S1_Beyond.pdf]

Supplementary Material S1.

The narrative methodological approach of interpreting Italian High Renaissance Paintings.

However, the naked breast is the index sign of Art nude. The art nude is an iconic sign of Western Art as visual culture (the artistic concept of nudity is a symbol of perfection and sin, and purity and shame as well). Raphael uses the female body (*signifiant*) to symbolize sensual beauty (*signifié*). *Significantly*, the woman in the painting reveals her left breast. Latin word "sinister" may signify sin, as Dante Alighieri mentions in many places of the *La Divina Commedia* that he and the Virgil go down to the left in the lower circles of Hell ("And he to me: "You know this place is round; and though the way that you have come is long, and always toward the left and toward the bottom, (…)", *Hell*, Song XIV, lines 124-126) [24]. As Maurizio Bettni notes, the nature of nude is dualistic. In the Roman Empire, exposing nude bodies outside domestic area was perceived as inappropriate. But truth is naked (Latin *nuda veritas*, *nuda simplicitas*) [25]. Symbolic function in Raphael's *La Fornarina* is related here to the expressive process, causing the sexual excitement of the viewer but also reflects intimate feelings of Raphael in historical context [1].

Ancient Greece is the birthplace of the beauty ideal. The epitome of perfection was an act, i.e., figures of athletes and gods of both sexes (see for example marble statue of Aphrodite of Syracuse in National Archeological Museum in Athens, Greece: https://www.aspectsofbeauty.gr/en/). Greeks cultivated the body, living in symbiosis with the nature and with the *logos* as a rational element that permeates the entire cosmos [25]. However, also in Greece, body nudity is ambiguous. In the hierarchy of being expressed by the philosopher Plato, the sensual things are only an imperfect reflection of the idea. Striving for perfect beauty, we are doomed to commune with poor beauty, contaminated by transience, disease, old age [26].

Medieval art inherits this ambivalence. Innocently naked in the act of divine creation, shamefully naked in human sin. Medieval theology developed four symbolic meanings of nudity. Nudity *naturalis* is the primal paradise innocence of the first people, tainted by their lust. Nudity *temporalis* characterizes various penitents (hermits, ascetics) who renounce temporal goods. Nudity *criminalis* is vicious and sinful. Nudity *virtualis* is simplicity, honesty, truth, opposed to appearances and superficiality [27].

Using the example of the female nude in Italian Renaissance art, Jones analyzes a specific way of showing women and men (naked, vain, passive, sexually tempting) as part of a spectacle designed to evaluate a man. The main protagonist of the act, the viewer in front of the painting (the external focalizer), is never painted. However, as a hidden viewer, it is for him that the figures assume the act's role [28].

The hidden position of the viewer towards the discovered status of women is a characteristic feature of European nude art. All the nude art presents voyeurism, a kind of cultural deviation. It involves cultivating the excitement of macho power passed on to the viewer, allowing him to

remain in control by looking at a distance and having a dominant position [29].

As mentioned above, nudity was a symbol of truth in traditional European art. Especially contrasted with its opposite, as in the painting by Titian *Sacred and Profane Love*, it symbolizes actual existence instead of its appearances. It was a symbol of internal or innate beauty (*pulchritudo innata*), grasped by reason, in opposition to artificial external embellishments (*ornamentum*) that appealed only to the senses. The human being was a part of the cosmos, and therefore the image should reflect his inner beauty and harmony. Harmony, proportion, and geometric symmetry were the artistic values [28]. Analysis of paintings belongs to the methodology of visual culture. The interpretation of a work of art is based on specific criteria, the disclosure of which is part of its justification. In addition to the traditional compositional interpretation, the critical methodology of visual studies refers to other interpretations such as semiology, discourse analysis, and psychoanalysis [30].

The references are in the References section of the main article and below as well:

1. Snell, M. Was Raphael Married? ThoughtCo. Available online: https://www.thoughtco.com/was-raphael-married-3969429 (accessed on 1 June 2025).

25. Bettini, M. In *Il nudo. Eros, natura, artificio*; Fossi, G., Ed.; Giunti Editore: Florence, Italy, 2019.
26. Plato. *The Allegory of the Cave*. Translated by Shawn Eyer. Plumbstone Books. Available online: https://scholar.harvard.edu/files/seyer/files/plato_republic_514b-518d_allegory-of-the-cave.pdf (accessed on 31 August 2025).
27. Poprzecka, M. *Przegląd Historyczny* **2009**, *100 (3)*, 363–385.
28. Jones, J. *The Loves of the Artists. Art and Passion in the Renaissance*. Simon & Schuster, Great Britain/United States of America, 2013.
29. Mulvey, L. Visual pleasure and narrative cinema. In *Film theory and criticism: Introductory readings.* G. Mast, G.; Cohen, M.; Braudy, L., eds. New York: Oxford University Press: New York, Unites States of America, pp. 746–757.
30. Rose, G. *Visual Metodology. An Introduction to the Interpretation of Visual Material*s (4th Ed.). SAGE Publications Ltd.: London, Great Britain, 2001.
