# Supplementary material for: Beyond the Image Frame: An Art-Based Pedagogical Framework for Teaching Diagnostic Reasoning in Breast Ultrasound to Medical Students
Source: Diagnostics (Basel). 2026 Feb 23;16(4):642. doi: 10.3390/diagnostics16040642 (PMC12939138; doi:10.3390/diagnostics16040642)
Supplement: Supplementary file 1 [file diagnostics-16-00642-s001.zip › Supplementary Material S2_Beyond.pdf]

Supplementary Material S2

"Be like the Raphael" –
breast ultrasound examination presentation

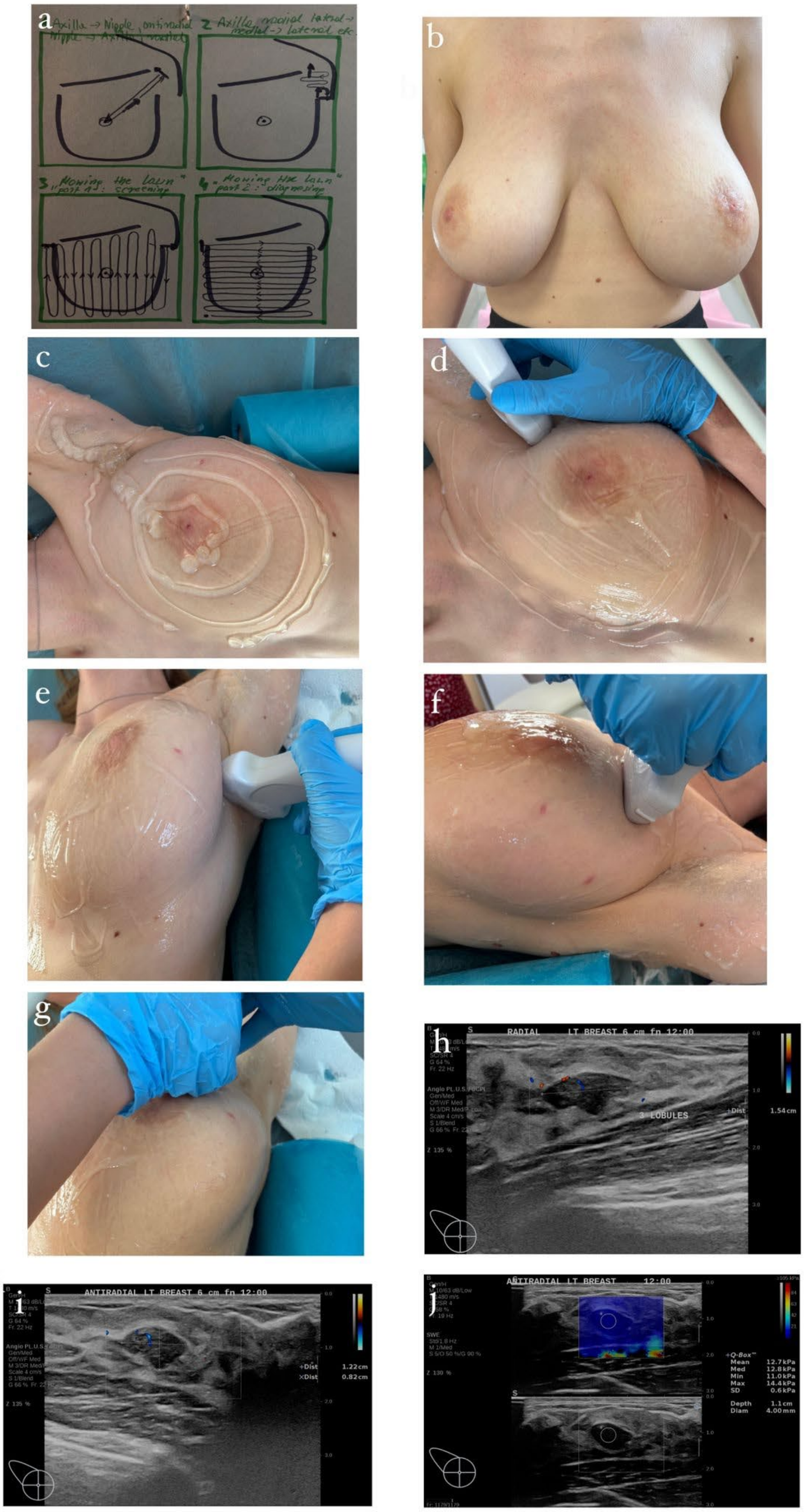

Figure S1. Panel documenting the breast ultrasound workshop entitled “Be like Raphael”; a) “cardboard” with a sketch showing the direction of the examination during breast ultrasound, b) appearance of the breast glands of the examined patient, c) preparation for the examination and application of gel to the skin of the breast, d) first movement of the probe towards the breast – probe radially in the Spence's tail location, e) determining the examination frame – probe at 3:00 o'clock at the radial border of the gland, f) probe positioned anti-radially (but held incorrectly), g) support when positioning the probe – radially, h) image of the lesion – B mode presentation, radial dimension, i) image of the focal lesion – B mode presentation with microflow imaging, anti-radial dimension, j) presentation of the focal lesion in elastography, anti-radial dimension; the examining hand of images f-g belongs to 5th MD student and co-author Julia Nowakowska.
