# Supplementary material for: Beyond the Image Frame: An Art-Based Pedagogical Framework for Teaching Diagnostic Reasoning in Breast Ultrasound to Medical Students
Source: Diagnostics (Basel). 2026 Feb 23;16(4):642. doi: 10.3390/diagnostics16040642 (PMC12939138; doi:10.3390/diagnostics16040642)
Supplement: Supplementary file 1 [file diagnostics-16-00642-s001.zip › Supplementary Material S3_Beyond.pdf]

Supplementary Material S3

Free interpretation of the image of *La Fornarina* by Students

1. Group A

Protocol text of the homework: It is worth noting here that young women also suffer from breast cancer. Despite the fact that the disease has not changed, today a young woman with BC is in a different medical and cultural situation where she is not alone with her problem, she communicates with medical staff at a distance via the Internet as the main communication channel and she is covered by professional care that was once dedicated only to people from the higher classes. Secondly, the image is a medium through which we share our experience - in this case, the experience of our illness. Our patient agreed to collage her reconstruction with *La Fornarina* in order to show that women should have her breasts examined even in young age during gynecological visit (Figure S3.1).

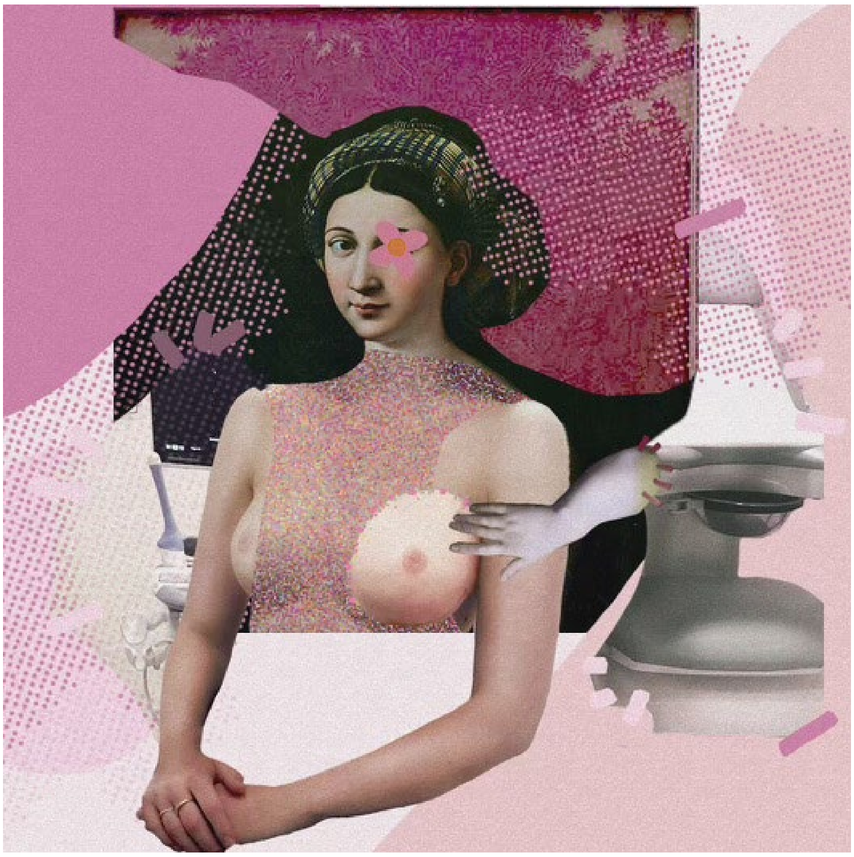

Figure S2. "Homework" result of Group 1; A collage of 20-years old patient after operating with breast cancer.

2. Group B

The domestic scenery of the photograph (Figure 10) brings attention to the issue to reliance on self-assessment in breasts' health monitoring. Time constrains, social stigma and other factors often discourage females from seeking professional opinion, and relying on their own. Lack of

specialist knowledge carries the risk of unintentional negligence and overlooking early signs of pathology.

The picture (Figure S3.2) emphasizes the need for more effective and widespread self-assessment tools. The phone pressed against the breast suggests the emerging research of smartphone-screening technologies. Such portable and accessible diagnostic devices may reduce the gap between professional diagnosis and casual examination, by enhancing accuracy of self-assessment. With the improvement of modern technology such techniques could improve the accuracy of self-assessment, thus improve the quality of health management among women.

Beyond the literal representation, the image further provokes deeper reflections regarding integration of medical technology into everyday life. Conducting self-assessment with digital tools in the privacy of a bathroom embodies such shift occurring in the developing world and encourages to make use of the possibilities if offers.

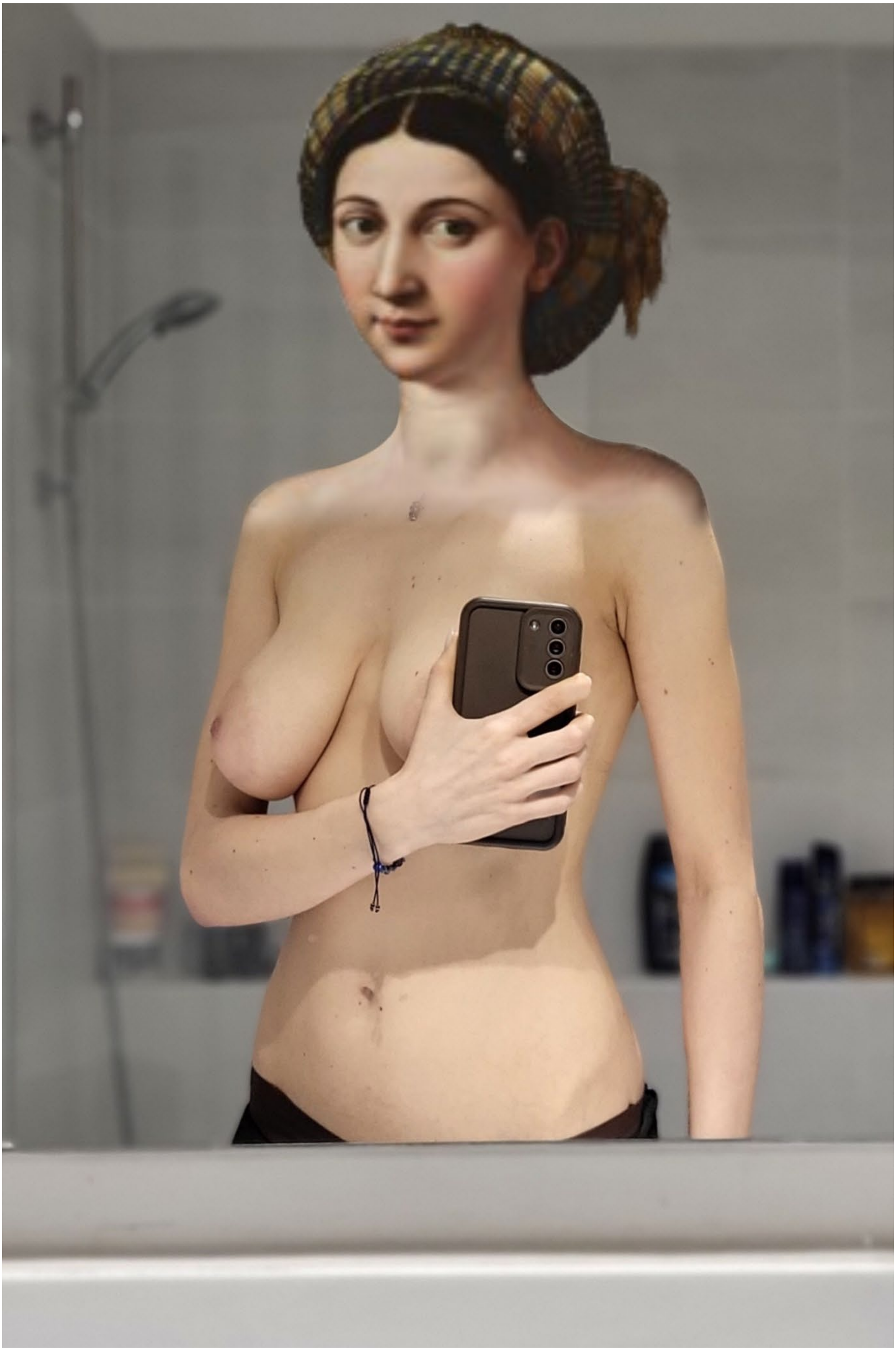

Figure S3. „A portrait of a young woman examining her breasts with a smartphone" (collage, with patient's permission).

3. Group C

The patient had been undergoing regular breast examinations for twenty years. It was a routine check for her, but a source of anxiety in the same time. The examinations repeatedly revealed abnormalities—cysts or benign lumps. She was always accompanied by fear, because there was a family history of cancer, and many people passed away shortly after diagnosis. After each ultrasound, she left the office with relief that lasted until her next visit.

This time it was different—she noticed swelling under her left armpit. She became concerned. It had been a year since her last checkup, so she decided to get tested as soon as possible. Unfortunately, she couldn't find a doctor or an available appointment in her town. She started to panic—she felt that "something was wrong, something was different than usual," but she had no way of checking it right away.

With her husband's help, she found a clinic in Gdańsk and made an appointment for a senological consultation. On her way to the appointment, she was overcome with paralyzing stress. During the examination, when the doctor expressed concern about the nature of the lesion, her world turned upside down. In an instant, all her plans—a new job, vacations with her children, and above all, her life—were thrown into question.

A biopsy was performed. Diagnosis: breast cancer. Is this a nightmare? A mistake? What now? Will I live? Many questions popped into her head—fear, panic, and a feeling of helplessness all at once.

And yet, something essential happened amid all this chaos. The patient was not left alone, as a team of specialists showed concern and took care of her health. Consultations, further tests, scheduling appointments, clear communication—all of this lifted her spirits, and her initial terror began to turn into calm. The team organized everything as quickly as possible so that she could recover from her illness.

Today, the patient has had a consultation. She is awaiting the results of genetic testing and surgery. She is aware of the treatment options available to her, depending on the results of further histopathological and genetic testing. She is afraid, but at the same time, she is calm. Time, efficient organization, and communication played a big role in this. Not only for the quick start of therapy, but also for the patient's mental well-being.

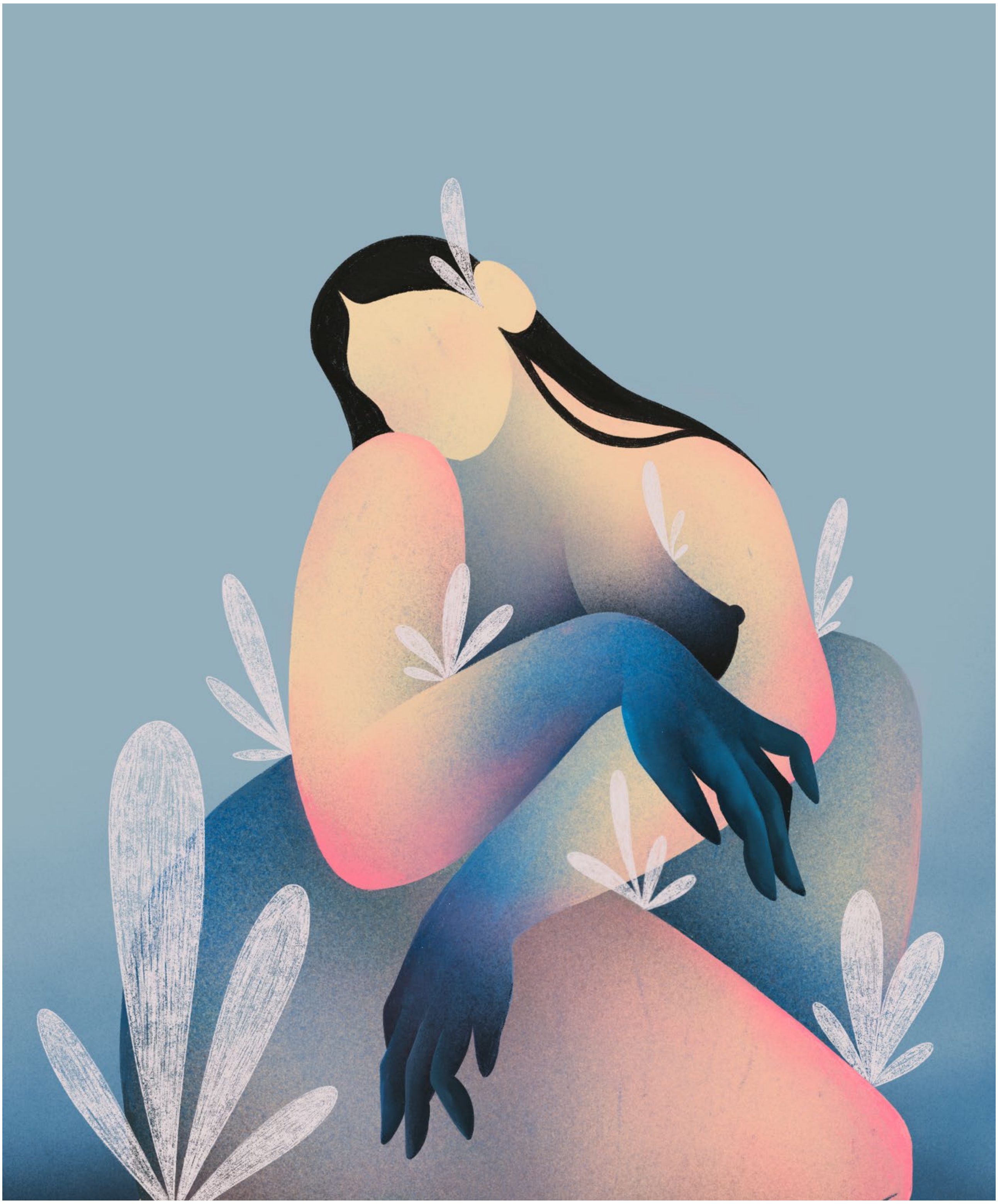

Figure S4. “Tenderness”.

4. Group D

Group D requested a letter in which the patient described the diagnosis process from her perspective (abbreviated, preserving the intent, consulted with the patient).

“In May 2025, when my husband noticed a lump in my breast, I felt tremendous fear, but I ignored it, hoping that the lesion would disappear.

Nevertheless, I couldn't shake my anxiety, so I started looking for a doctor to examine me. Based on the best reviews, I chose Dr. Marcin Śniadecki, who, after a thorough examination, concluded that the lump was suspicious. I received a BI-RADS 4C result, which meant minimum 50% risk of malignancy. Further tests, including a mammogram [actually tomosynthesis - note by Group D], gave a BI-RADS 5 result, which further deepened my fears. I felt increasingly depressed and desperate. However, I still held on to the hope that maybe it wouldn't be so bad. The doctor referred me for a biopsy, but I already knew that only a miracle could change the result. I informed my family, although I understood that they would be much more affected by this than I was. Every phone call, every question deepened my pain, but the support of my loved ones gave me strength. After two weeks, the doctor gave me the result – cancer, but one of the milder types – I felt a huge relief, because it could have been much worse. I decided to have a mastectomy based on my intuition and trust in my doctor. During this difficult time, I realized how important it is to find a doctor who not only treats you, but also understands and supports you. Now I trust doctors to help me through the next stages of treatment.”

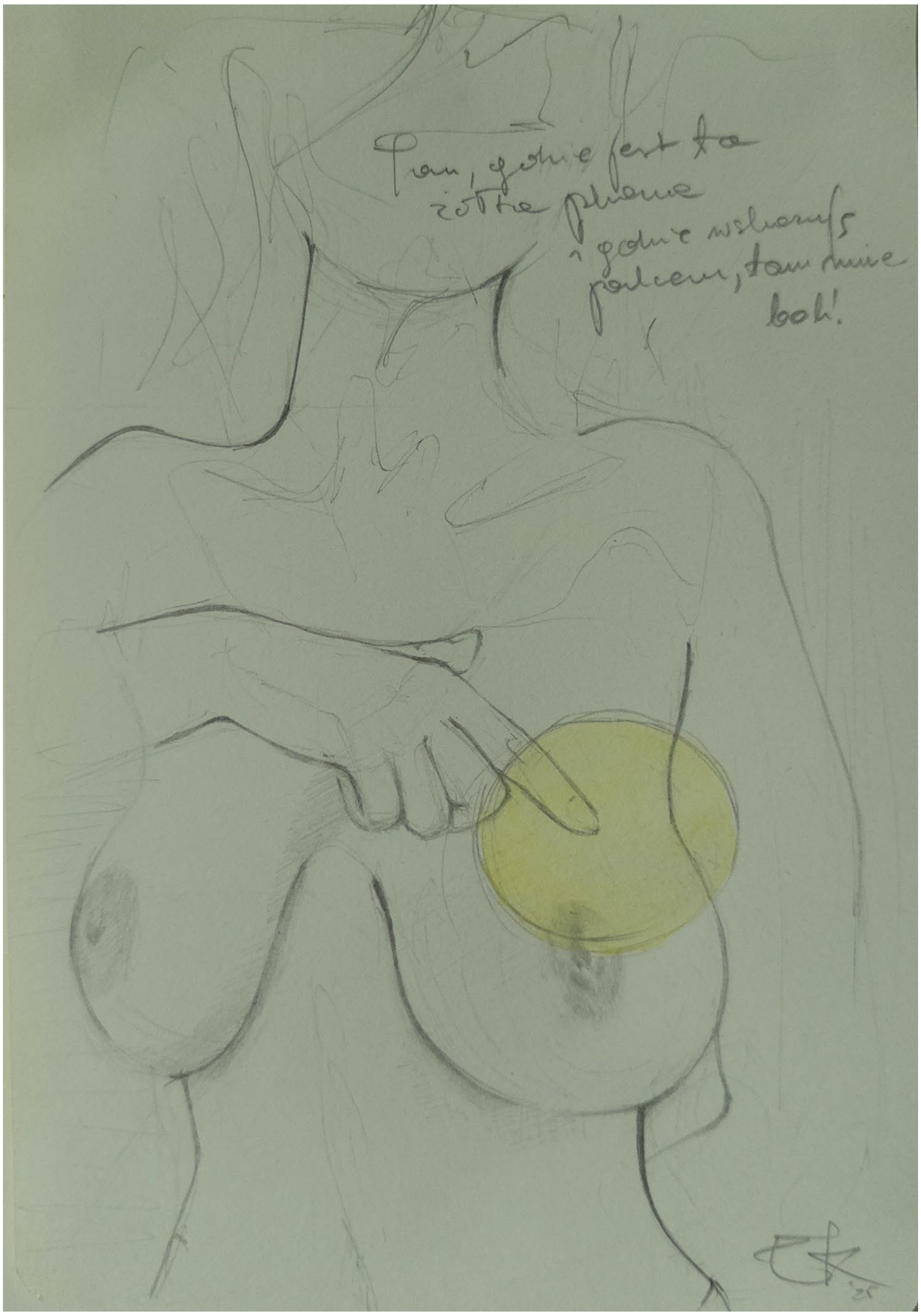

Figure S5. "Auto-Mammae"; The translated Polish inscription into English: "There, where the yellow spot is located, and where I point my finger, there it hurts".
